# Light-Assisted Fabrication of Hierarchical Azopolymer Structures Using the Breath Figure Method and AAO Templates

**DOI:** 10.1021/acs.langmuir.4c02410

**Published:** 2024-07-16

**Authors:** Ming-Hsuan Chang, Lin-Ruei Lee, Meng-Ru Huang, Tsung-Hung Tsai, Yi-Fan Chen, Yu-Ting Hong, Yu-Chun Liu, Jiun-Tai Chen

**Affiliations:** † Department of Applied Chemistry, National Yang Ming Chiao Tung University, Hsinchu 300093, Taiwan; ‡ Center for Emergent Functional Matter Science, National Yang Ming Chiao Tung University, Hsinchu 300093, Taiwan

## Abstract

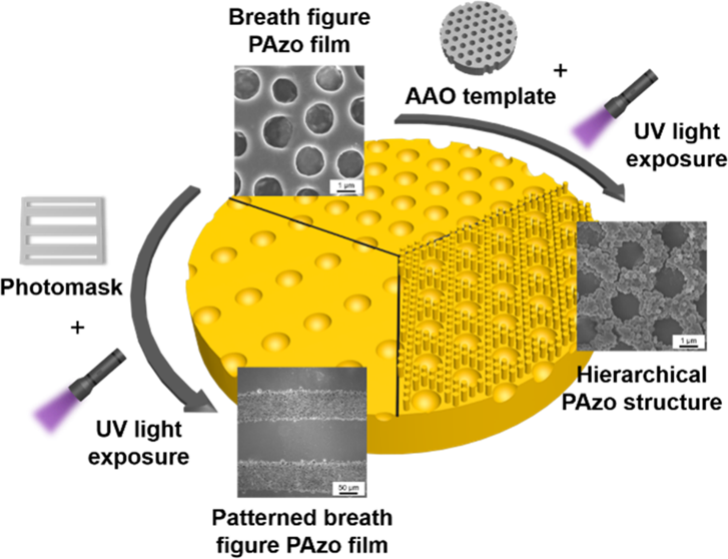

Hierarchical polymer structures have garnered widespread application across various fields owing to their distinct surface properties and expansive surface areas. Conventional hierarchical polymer structures, however, often lack postfabrication scalability and spatial selectivity. In this study, we propose a novel strategy to prepare light-assisted hierarchical polymer structures using azopolymers (PAzo), the breath figure method, and anodic aluminum oxide (AAO) templates. Initially, the breath figure PAzo films are prepared by dripping a PAzo chloroform solution onto glass substrates in a high-humidity environment. The AAO templates are then placed on the breath figure PAzo film. Upon ultraviolet (UV) light exposure, the azobenzene groups in the azopolymers undergo *trans–cis* photoisomerization. This process causes the glass transition temperature (*T*_g_) of the PAzo to become lower than room temperature, allowing the azopolymer to enter the nanopores of the AAO templates. The hierarchical azopolymer structures are then formed by using a sodium hydroxide solution to remove the templates. Furthermore, exploring the effects of PAzo concentration and UV light exposure duration on the film morphology reveals optimized conditions for hierarchical structure formation. Additionally, the water contact angles of these polymer structures are measured. The hierarchical PAzo structures exhibit higher hydrophobicity compared with the flat PAzo films and the PAzo breath figure films. Finally, patterned breath figure films can be prepared using designed photomasks, demonstrating the method’s capability for spatial selectivity.

## Introduction

Hierarchical polymer structures, known for their multifaceted length scales and unique surface properties, offer numerous advantages across biomedical, environmental remediation, and materials science domains.^1−3^ Conventional hierarchical polymer structures, however, suffer from limitations in postfabrication size scale adjustments and spatial selectivity. In this study, we present a novel methodology for fabricating light-assisted hierarchical polymer structures by integrating azopolymer (PAzo), the breath figure method, and anodic aluminum oxide (AAO) templates. PAzo is a type of polymer material that exhibits unique photoresponsive properties.^4^ Upon exposure to specific light wavelengths, the polymer undergoes *trans–cis* isomerization, leading to alterations in physical and chemical attributes such as surface hydrophilicity/hydrophobicity, optical properties, and glass transition temperature (*T*_g_). Therefore, PAzo finds applications in various fields, including optics, nanotechnology, and biotechnology.^5−7^

The breath figure method, a widely employed technique in polymer science, facilitates the creation of ordered porous structures through the self-assembly of water droplets on a polymer film surface.^8−10^ This method capitalizes on the natural dewetting behavior of polymers in the presence of water vapor, resulting in the formation of regular arrays of pores. The breath figure method offers several advantages, including simplicity, cost-effectiveness, and time efficiency.^11^ Moreover, by carefully controlling experimental parameters such as temperature, humidity, and polymer concentration, it is possible to adjust the size and properties of the pores.^12^

There are several common methods for fabricating polymer nanoarrays, including photolithography, the nanoimprinting method, and the template method.^13−15^ While photolithography and the nanoimprinting method are effective, they come with inherent limitations. Photolithography demands complex equipment and precise light exposure control, leading to high costs and technical challenges. Similarly, the nanoimprinting method involves expensive molds and meticulous alignment processes, thereby adding complexity and expense. In contrast, the template method is widely preferred because of its simplicity and cost-effectiveness. Among template methods, AAO templates stand out for their large-area and highly regular nanopores achieved through a two-step anodization process.^16^ Additionally, AAO templates offer precise control over pore size and template thickness by adjusting electrolytes or anodization duration and can be selectively removed using weak acids or bases, making them the most utilized templates.^17,18^

Several techniques are available for introducing polymers into the nanopores of AAO templates, including the solution-wetting method, the melt-wetting method, the solvent-vapor annealing wetting method, and the light-induced nanowetting method.^19−23^ In the solution-wetting method, polymers are dissolved in solvents and the resulting solutions are employed to infiltrate the nanopores. In the melt-wetting method, polymers are heated beyond their melting point (*T*_m_) or glass transition temperature (*T*_g_) to aid penetration into the nanopores. In the solvent-vapor annealing wetting method, polymers are exposed to solvent vapors, causing polymer chain swelling and facilitating pore infiltration. Lastly, in the light-induced nanowetting method, PAzo undergoes *trans–cis* transformation upon exposure to specific light wavelengths, allowing infiltration into nanopores if the *T*_g_ of the *cis* form is lower than room temperature.

In this work, we integrate PAzo, the breath figure method, and AAO templates to fabricate hierarchical polymer structures. Following PAzo synthesis through a four-step process, porous PAzo films are obtained using the breath figure method. Nanostructures are subsequently formed on the breath figure PAzo films using the light-induced nanowetting method, enabling PAzo infiltration into AAO templates and resulting in hierarchical polymer structures. Furthermore, we explore the effects of different concentrations of PAzo solution and ultraviolet (UV) light exposure durations on the morphologies of the breath figure PAzo films, revealing that a 4 wt % PAzo solution yields the most regular pore size and arrangement, with pore area increasing with prolonged light exposure. Additionally, through measurements of water contact angles, we observe that the breath figure PAzo film exhibits greater hydrophobicity compared with the flat PAzo film, with hydrophilicity increasing with longer light exposure. Moreover, the hierarchical PAzo structure is the most hydrophobic among all the structures studied. Lastly, we demonstrate the fabrication of patterned breath figure films using variously shaped photomasks.^24−27^ This innovative method not only showcases expanded applications of hierarchical polymer structures but also enhances their versatility.

## Results and Discussion

**Figure 1:**
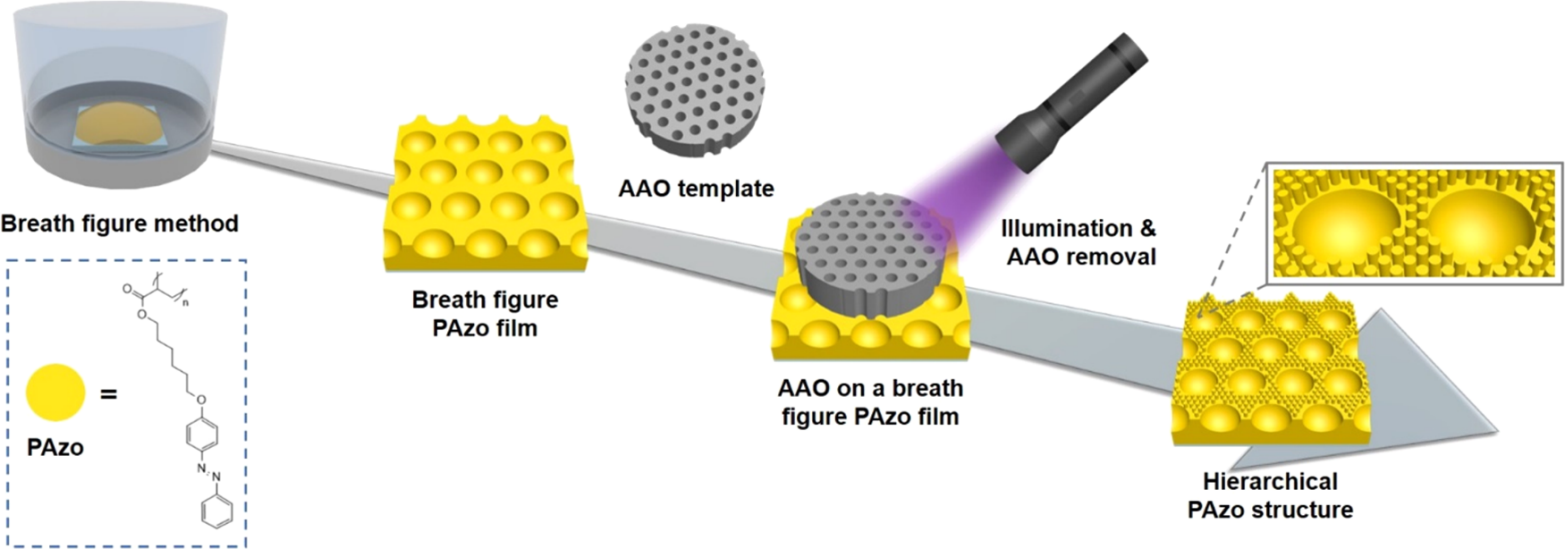
Schematic diagram of the experimental processes to fabricate the breath figure PAzo films, followed by the light-induced nanowetting method to create the hierarchical PAzo structures.

Figure 1 depicts the schematic diagram detailing the experimental processes to fabricate the hierarchical PAzo structures. Initially, a 4 wt % PAzo solution in chloroform is prepared and deposited onto a glass substrate via drop-casting. Subsequently, the sample is placed in a sealed container under high humidity conditions at 30 °C, allowing the self-assembly arrangement of water droplets formed via water vapor condensation on the surface of the PAzo film. This process is followed by the evaporation of the solvent, resulting in the solidification of the film and the formation of the breath figure PAzo film. An AAO template is then positioned atop the breath figure PAzo film. Upon UV light irradiation, the *trans*-PAzo film is converted into *cis*-PAzo with a *T*_g_ below room temperature, facilitating its infiltration into the nanopores of the AAO templates through capillary force. Subsequent irradiation with visible light solidifies the PAzo, resulting in the formation of nanopillars within the nanopores of the AAO templates. Upon removal of the AAO template, the hierarchical PAzo structures are obtained.

**Figure 2:**
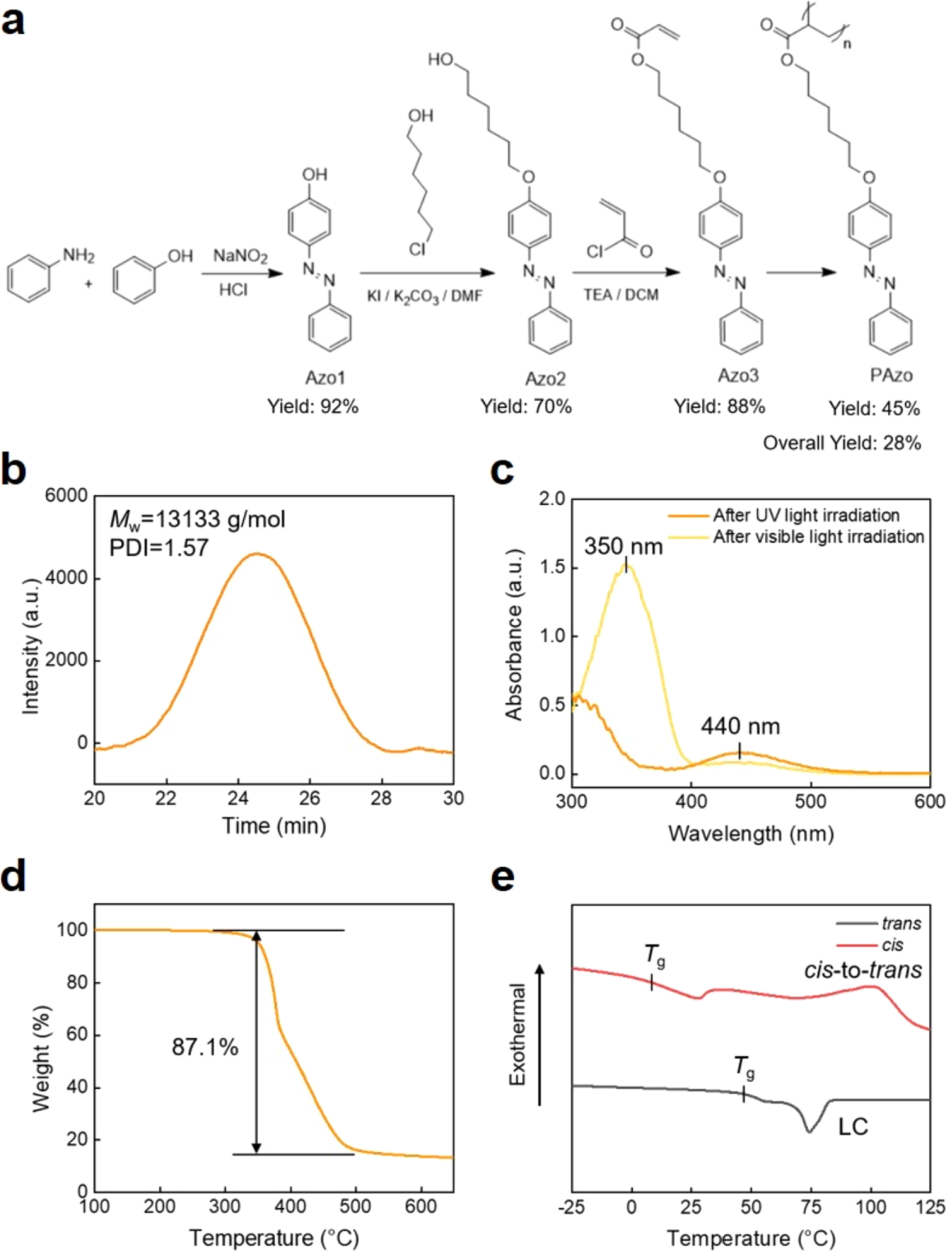
(a) Synthetic scheme of the PAzo. (b) GPC data of the PAzo. (c) UV–vis spectra of the PAzo after UV light irradiation and subsequent visible light exposure. (d) TGA data of the PAzo. (e) DSC data of the *cis*-form and *trans*-form PAzo.

Figure 2a illustrates the synthetic pathway of the PAzo, which involves four distinct steps. Initially, the azobenzene group (Azo1) is synthesized via a diazo-reaction and an azo coupling reaction employing aniline and phenol, respectively. Subsequently, Azo2 is formed through an S_N_2 reaction, which extends the carbon chains by six carbons. In the third step, Azo2 undergoes esterification to introduce a double bond (Azo3), facilitating subsequent polymerization. Finally, a free radical polymerization is employed to synthesize the PAzo. The molecular structure of the PAzo is confirmed using ^1^H nuclear magnetic resonance (NMR) spectrum, as depicted in Figure S1.

Gel permeation chromatography (GPC) analysis demonstrates that the weight-average molecular weight (*M*_w_) of the PAzo is 13133 g/mol with a polydispersity index (PDI) of 1.57, as illustrated in Figure 2b. Ultraviolet–visible (UV–vis) spectra, depicted in Figure 2c, demonstrate the photoisomerization behavior of the PAzo. Upon exposure to UV light (365–375 nm) for 10 min, the *trans*-PAzo converts to the *cis*-form; subsequently, upon irradiation with visible light (520–530 nm), the *cis*-PAzo transitions back to the *trans*-form. Thermal gravimetric analysis (TGA) data, presented in Figure 2d, indicates that PAzo begins to decompose at ∼285.4 °C. Differential scanning calorimetry (DSC) data, shown in Figure 2e, reveals that the glass transition temperature (*T*_g_) of the *trans*-PAzo is ∼53.5 °C, indicating a solid state at room temperature. Conversely, the *T*_g_ of the *cis*-PAzo is ∼17.1 °C, indicating a liquid-like state at room temperature.

**Figure 3:**
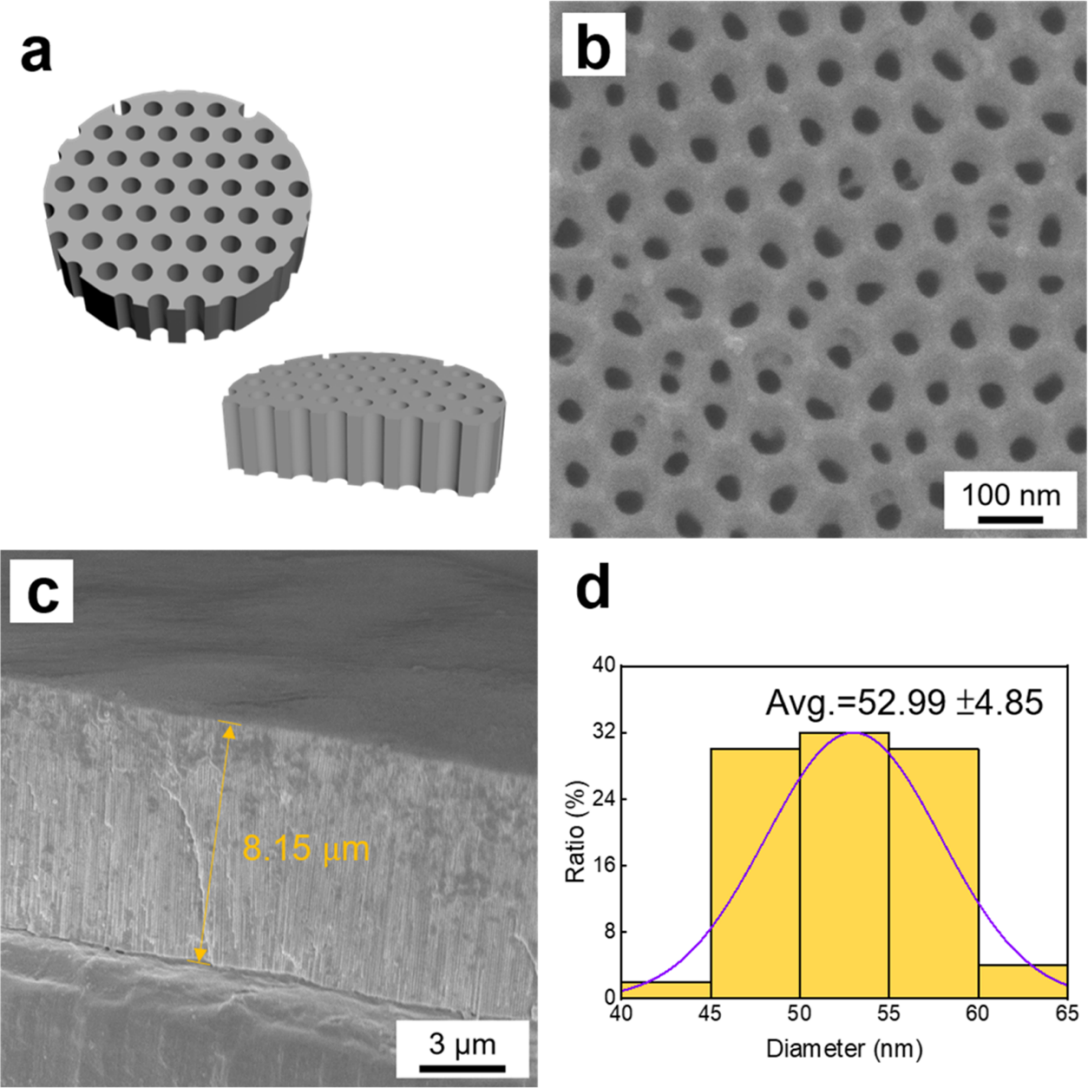
(a) Graphical illustrations of the top view and cross-sectional view of an AAO template. (b, c) SEM images of the top view and cross-sectional view of an AAO template: (b) top view and (c) cross-sectional view. The thickness of the AAO template is also indicated. (d) Corresponding diameter distributions of the nanopores within an AAO template.

In this study, AAO templates are utilized to infiltrate the PAzo through capillary force. Subsequent removal of the templates via sodium hydroxide solution allows for the release of PAzo nanostructures. Graphical illustrations and scanning electron microscopy (SEM) images of the top view and cross-sectional view of an AAO template are presented in Figure 3a–c. The AAO templates are fabricated using a two-step anodization method. The duration of the first anodization step primarily controls the regularity of the AAO templates while the duration of the second anodization step determines their thicknesses. Additionally, the pore sizes of the AAO templates can be adjusted by varying the electrolytes and pore-widening durations. The cross-sectional view of an AAO template, with a thickness of ∼8.15 μm, is depicted in Figure 3c. Figure 3d displays the corresponding diameter distribution of nanopores within an AAO template, with an average pore diameter of ∼53 nm.

**Figure 4:**
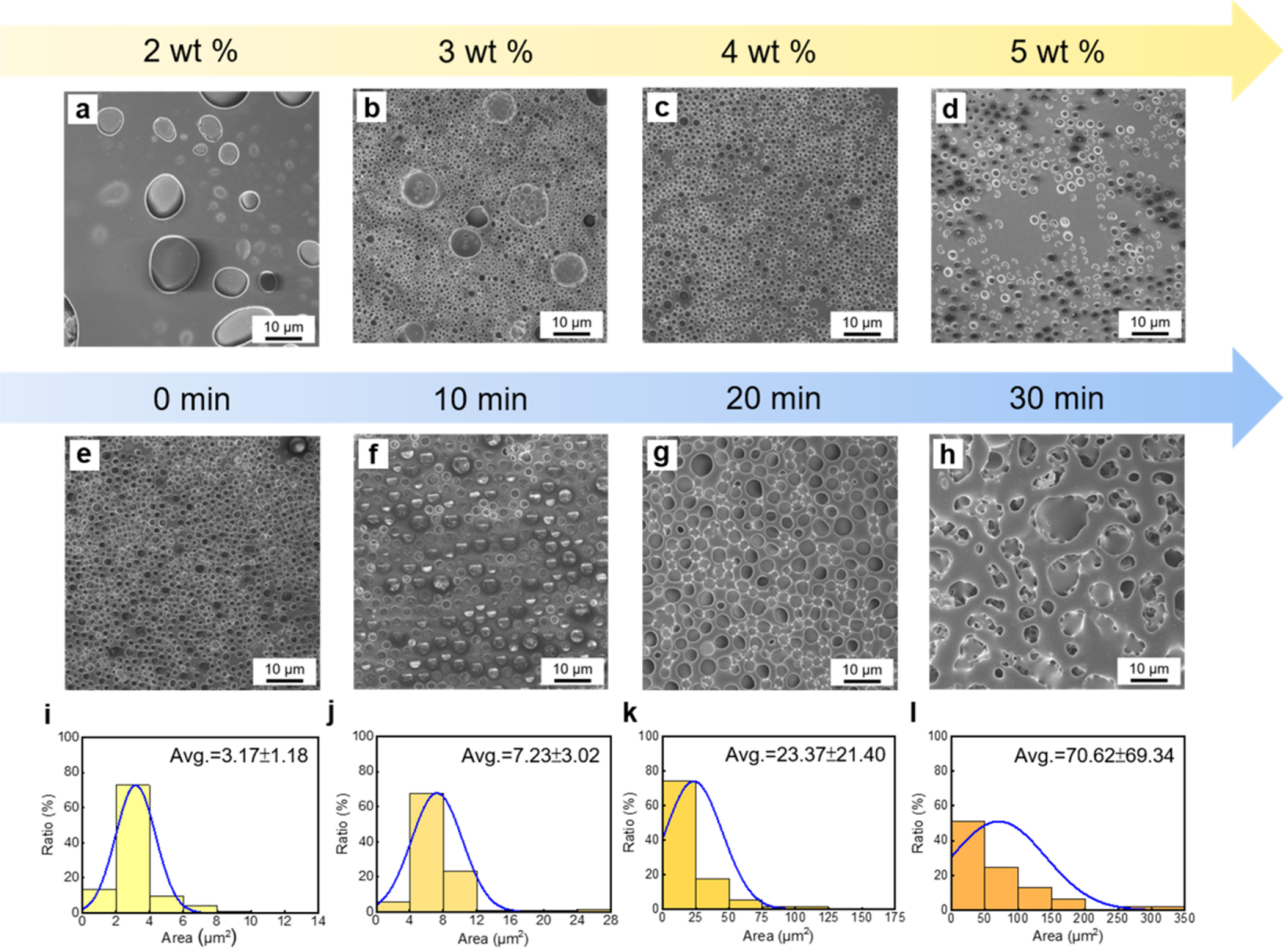
(a–d) SEM images of the breath figure PAzo films at varying concentrations: (a) 2, (b) 3, (c) 4, and (d) 5 wt %. (e–h) SEM images of the breath figure PAzo films under different illumination durations: (e) 0, (f) 10, (g) 20, and (h) 30 min. (i–l) Corresponding pore area distributions of the breath figure PAzo films under different illumination durations: (i) 0, (j) 10, (k) 20, and (l) 30 min.

The breath figure PAzo films are fabricated through drop-casting a PAzo solution in chloroform onto a glass substrate under high humidity. We first explore the impact of concentration on the morphology of the breath figure PAzo films, as depicted in Figure 4a–d. The concentration of PAzo plays a crucial role in determining the morphology of the breath figure polymer films. At lower PAzo concentrations, there is not enough polymer to form a stable and continuous film, resulting in less ordered pores. Conversely, at higher PAzo concentrations, the solution becomes too viscous, which hinders the evaporation of the solvent and the self-assembly of water droplets, leading to some areas where pores fail to form. In the case of a 2 wt % PAzo solution (Figure 4a), the pores of the breath figure PAzo film exhibit uneven areas and distributions. In the case of a 3 wt % solution (Figure 4b), although the pores are evenly distributed, some excessively large pores are still present. With a 4 wt % solution (Figure 4c), however, the pores display uniform areas and distributions. While the pore areas are uniform in the case of a 5 wt % solution, the excessively thick film leads to some areas where pores fail to form. Consequently, we opt for a 4 wt % solution as the experimental condition for subsequent experiments.

Due to the phenomenon of photoliquefaction of PAzo upon exposure to UV light, we further investigate the effect of the illumination duration on the pore area of the breath figure PAzo films. After preparing the breath figure PAzo film, we expose it to UV light for different durations to induce light-induced liquefaction of PAzo, followed by illumination with visible light to revert the liquid-like state of *cis*-PAzo back to the solid state of *trans*-PAzo in the breath figure PAzo film again. A longer illumination time indicates a longer duration of polymer liquefaction. SEM images of the breath figure PAzo films under different illumination durations are shown in Figure 4e–h, accompanied by the corresponding pore area distributions in Figure 4i–l. Before exposure to UV light, the pore area of the breath figure PAzo film is ∼3.17 μm^2^ (Figure 4i). After 10 min of UV light exposure, the pore area increases to ∼7.23 μm^2^ (Figure 4j). With 20 min of UV light exposure, the pore area further enlarges to ∼23.37 μm^2^ (Figure 4k). Following 30 min of UV light exposure, the pore area significantly expands to ∼70.62 μm^2^ (Figure 4l). Figure S2 shows the cross-sectional view SEM images of breath figure PAzo films. These results indicate that, as the illumination duration increases, areas outside the pores gradually collapse along the periphery of the pores, the pore area of the breath figure PAzo film gradually increases, and eventually, the pores begin to merge, forming larger and fewer pores.

**Figure 5:**
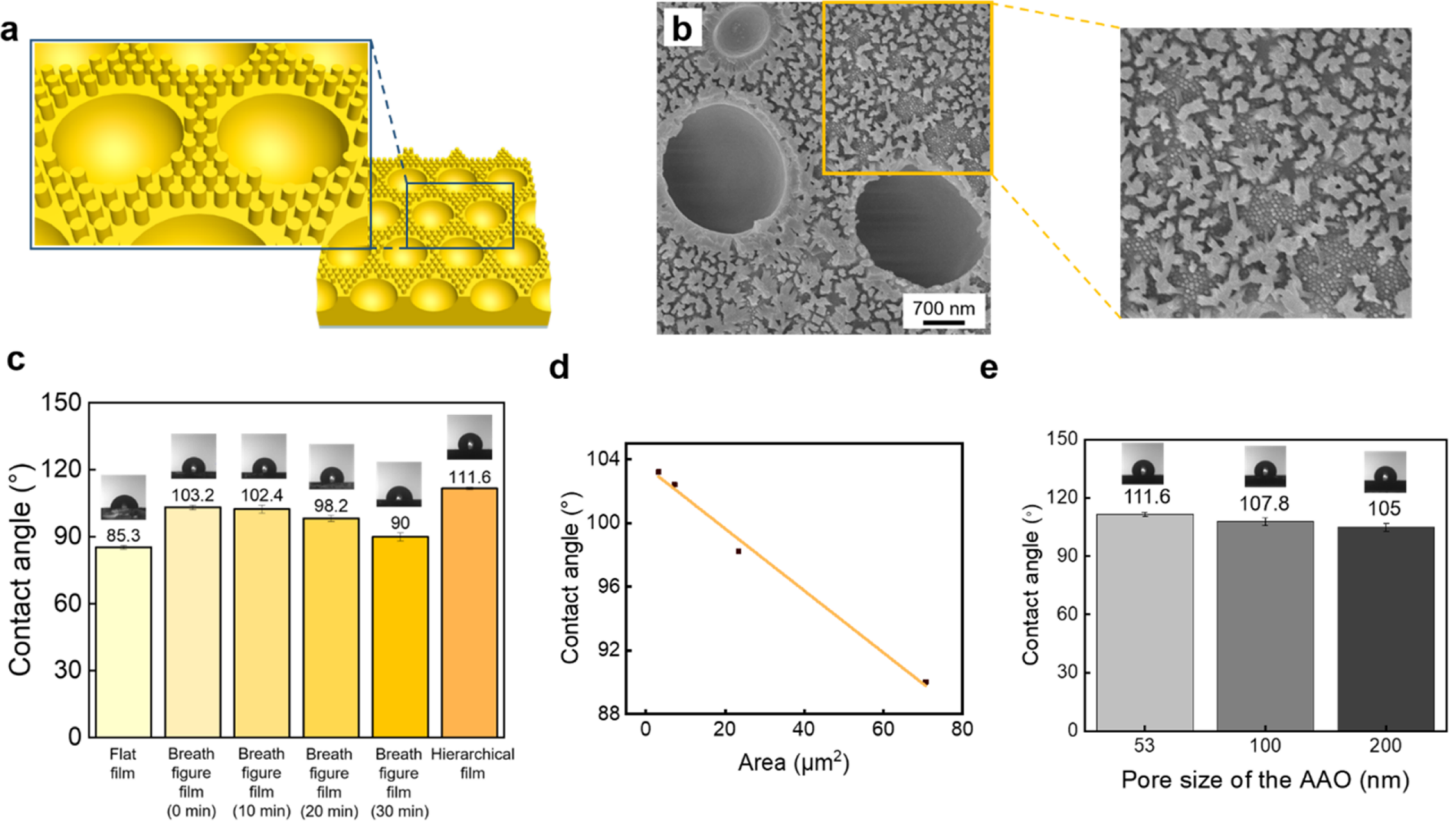
(a) Graphical illustration of the hierarchical PAzo structure. (b) SEM image of the hierarchical PAzo structure. (c) Plots of water contact angles for PAzo films, breath figure PAzo films under various illumination durations, and hierarchical PAzo structures. (d) Plots of water contact angles for the breath figure PAzo films with different pore sizes. (e) Plot of water contact angles for hierarchical PAzo structures fabricated using AAO templates with different pore sizes: 53, 100, and 200 nm.

In the light-induced nanowetting method, the breath figure PAzo film can be infiltrated into the nanopores of the AAO template upon UV light irradiation, followed by the removal of the AAO templates using a sodium hydroxide solution to obtain the hierarchical PAzo structures. As the UV irradiation time increases, the height of the nanostructures increases. The AAO template does not absorb light in the 365–375 nm range, as shown in Figure S3. The use of NaOH(aq) effectively removes AAO templates without affecting the surface structures of PAzo. Figure 5a,b present the graphical illustration and SEM image of the hierarchical PAzo structure, respectively. The nanostructures tend to align with the AAO template pores, forming a hexagonal arrangement. Figure 5c illustrates the water contact angles of the PAzo samples with different morphologies. In flat PAzo films, the water contact angle is ∼85°. In the breath figure PAzo film, the water contact angle increases to ∼103°. With an increase in illumination duration, however, the water contact angle gradually decreases. Specifically, it changes from 103° before illumination to 102° after 10 min of illumination, 98° after 20 min, and 90° after 30 min. This observation indicates that, as the illumination duration increases, the breath figure PAzo film becomes more hydrophilic owing to the increased fluidity and smoothness of the surface. Furthermore, upon introduction of the AAO template into the breath figure PAzo film, the water contact angle of the hierarchical PAzo structure increases to ∼112°, indicating greater hydrophobicity compared with the breath figure PAzo film. The Cassie–Baxter model provides a theoretical framework to understand how hierarchical structures influence surface wettability. According to the Cassie–Baxter equation
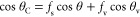
1where θ_C_ is the apparent contact angle, *f*_s_ is the fraction of the solid surface area, *f*_v_ is the fraction of the air surface area, θ is the intrinsic contact angle of the liquid on the solid, and θ_v_ is the contact angle of the liquid on air (180°). When the surface morphology is transformed into a hierarchical structure, the surface roughness increases, creating more air pockets. This result increases the fraction of the surface area that is composed of air (*f*_v_) and decreases the fraction of the surface area that is solid (*f*_s_). The increased number of air pockets significantly contributes to a higher value of *f*_v_. Given that θ*_v_* is 180° and cos 180° = −1, the term *f*_v_ cos θ_v_ becomes significantly negative. This negative contribution increases the overall apparent contact angle (θ_C_), making the surface more hydrophobic. Figure 5d demonstrates the changes in water contact angles corresponding to different illumination durations of the breath figure PAzo film with varying pore sizes. As the pore size increases, the water contact angle decreases, indicating higher hydrophilicity. Figure 5e shows that, as the pore size of the AAO template increases from 53 nm (112°) to 100 nm (108°) and then to 200 nm (105°), the water contact angle decreases. This observation indicates that larger pore sizes tend to increase the hydrophilicity of the hierarchical PAzo structures. This result is because larger pores allow more liquids to penetrate and spread on the surface.

**Figure 6:**
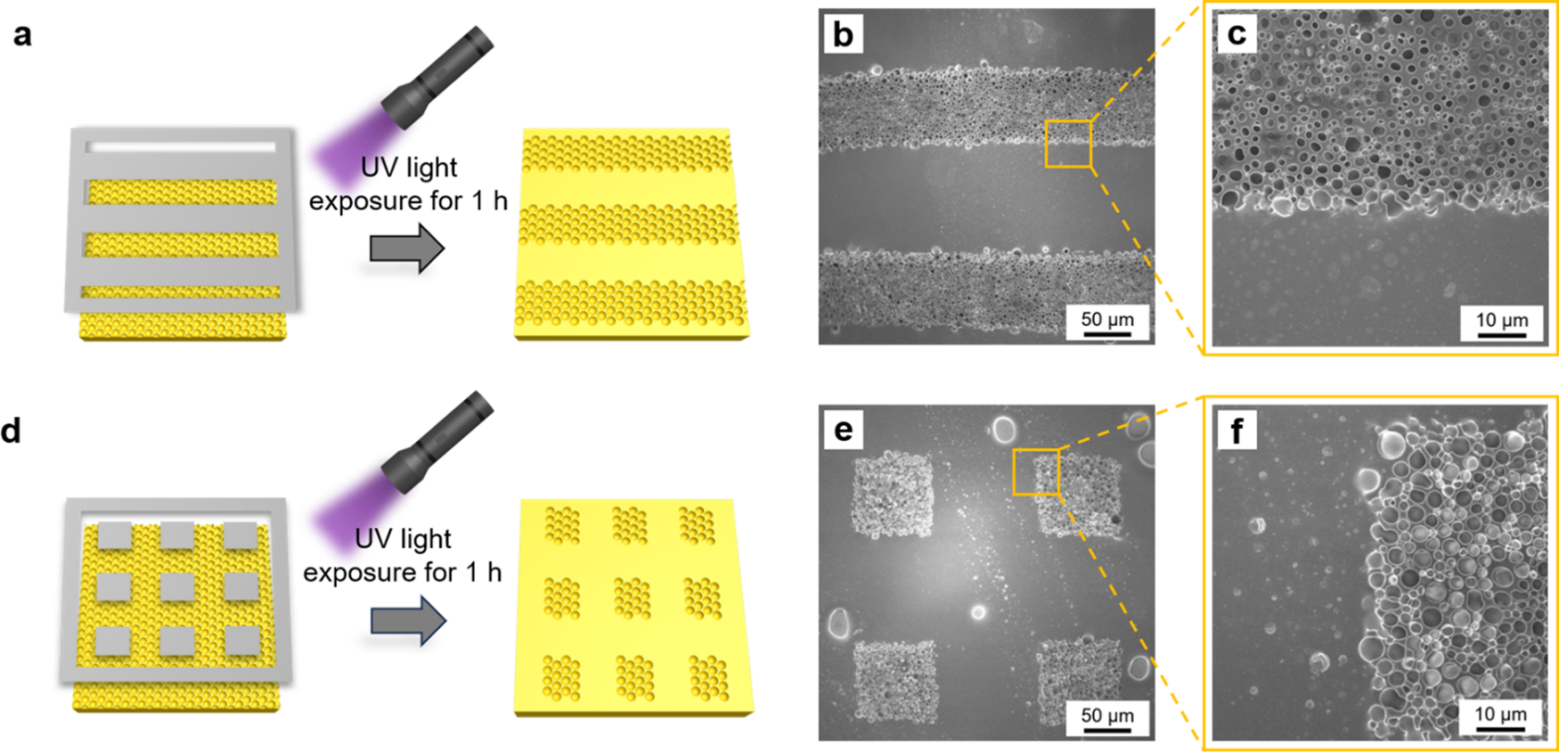
(a) Graphical illustration of the patterned breath figure PAzo film using a stripe-type photomask. (b, c) Low and high magnification SEM images of the stripe-patterned breath figure PAzo film. (d) Graphical illustration of the patterned breath figure PAzo film using a square-type photomask. (e, f) Low and high magnification SEM images of the square-patterned breath figure PAzo film.

In Figure 6, the generation of patterned breath figure PAzo films by harnessing the photoresponsive property of PAzo is demonstrated. By employing stripe-type and square-type photomasks, we generate stripe-patterned and square-patterned breath figure PAzo films, respectively. Figure 6a presents a graphical illustration of the stripe-patterned breath figure PAzo film irradiated by UV light using a stripe-type photomask. The stripe-type photomask consists of clear and dark regions, each with widths of ∼100 μm. After the breath figure PAzo sample is exposed to UV light for 1 h, the resulting stripe-patterned breath figure PAzo film is obtained, as illustrated in Figure 6b,c. Regions unaffected by UV light maintain the breath figure PAzo morphology while regions exposed to UV light undergo photoliquefaction, transforming into flat PAzo regions. The average widths of the breath figure PAzo regions and flat PAzo regions are ∼92 and 108 μm, respectively, closely matching the dimensions of the clear and dark regions of the stripe-type photomask.

Similarly, Figure 6d illustrates a graphical illustration of the square-patterned breath figure PAzo film irradiated by UV light using a square-type photomask. The widths of clear and dark regions of the square-type photomask are also ∼100 μm. Following exposure to UV light for 1 h, the square-patterned breath figure PAzo film is obtained, as depicted in Figure 6e,f. The average widths of the breath figure PAzo regions and flat PAzo regions are ∼93 and 108 μm, respectively, also closely matching the dimensions of the clear and dark regions of the square-type photomask.

## Conclusions

In this study, we introduce a novel methodology for fabricating light-assisted hierarchical polymer structures by integrating azopolymer (PAzo), the breath figure method, and anodic aluminum oxide (AAO) templates. Initially, the PAzo breath figure films are prepared by dripping a PAzo chloroform solution onto glass substrates in a high-humidity environment. The AAO templates are then placed on the PAzo breath figure film. Upon UV light exposure, the azobenzene groups in the azopolymers undergo *trans–cis* photoisomerization, allowing the PAzo to enter the nanopores of the AAO templates. After the removal of the templates, the hierarchical azopolymer structures are obtained. We then investigate the effects of different experimental parameters, including the PAzo concentration and UV light exposure duration, on optimizing the fabrication process for desired morphologies. Moreover, we analyze the surface properties of the PAzo structure with different morphologies using water contact angle measurements. The hierarchical PAzo structures exhibit the highest hydrophobicity. Furthermore, the demonstration of the patterned breath figure PAzo films using custom-designed photomasks underscores the method’s capability for spatial selectivity and expanded versatility. Through this method, we overcome the limitations of conventional hierarchical PAzo structures in postfabrication size scale adjustments and spatial selectivity, offering a feasible and versatile approach to fabricating hierarchical polymer structures. Furthermore, the light-controlled hierarchical polymer structures hold significant potential in various applications, including smart membranes for size-selective filtration. By leveraging the ability to modulate the pore size of the polymer structures through light exposure, these membranes could find applications in fields such as water purification, biomedical separations, and environmental remediation.

## Experimental Section

### Materials

Aluminum foils (99.9997%) were purchased from Alfa Aesar. Isopropanol (99.5%), acetone (99%), and ethanol (99.5%) were acquired from Echo Chemical. Phenol (>99%), sodium nitrite (99%), hydrochloric acid (>37%), potassium carbonate (99.995%), potassium iodide (>99.5%), perchloric acid (60–62%), chloroform (>99%), and sodium hydroxide were obtained from Sigma-Aldrich. Aniline (99%), anisole (99%), acryloyl chloride (96%), and potassium dichromate (99%) were sourced from Alfa Aesar. Phosphoric acid (95%) and oxalic acid were procured from Showa. 6-Chloro-1-hexanol was obtained from Nova Materials. 2,2′-Azobis(2-methylpropionitrile) (AIBN) (99%) was purchased from Aencore. Microscope glass slides were obtained from DGS. The weight-average molecular weight (*M*_w_) of the synthesized PAzo is 13,133 g/mol with a polydispersity index (PDI) of 1.57.

### Polymerization of the Azopolymer

The PAzo was synthesized via free-radical polymerization. Initially, azobis(isobutyronitrile) (AIBN) (30 mg) and the azobenzene-containing monomer (1.5 g) were dissolved in anisole (4 mL). Subsequently, the solution was connected to a vacuum system and subjected to 5 freeze–pump–thaw cycles. The solution was then heated to 80 °C and stirred for 24 h. Following polymerization, the solution was cooled to room temperature and precipitated in methanol (40 mL). The resulting precipitate was dissolved in tetrahydrofuran and added dropwise into methanol multiple times to remove any unreacted monomer. The resulting PAzo was then collected and dried in an oven at 40 °C for 24 h to remove residual solvents. The polymerization yield was ∼45%. ^1^H NMR (CDCl_3_): δ (ppm) = 7.76–7.92, 7.42, 6.9 (for protons on the azobenzene group), 3.8–4.12 (Ar–O–CH_2_, – O–CH_2_), 2–0.8 (for protons on the polymer backbone and – CH_2_–CH_2_–CH_2_–CH_2_−).

### Fabrication of the AAO Templates

The AAO templates were created using a two-step anodization method.^28^ Initially, an aluminum foil was cut into sheets with the size of 5 × 1.5 cm^2^. The sheets underwent ultrasonic oscillation in isopropanol, acetone, and deionized (DI) water for 10 min, sequentially. Subsequently, electropolishing was performed at 20 V using a solution consisting of 80 vol % ethanol and 20 vol % perchloric acid at 0 °C for 120 s. After rinsing with DI water, the sheets were anodized at 0 °C and 40 V in a 0.3 M oxalic acid solution for 4 h. Following this process, a chemical etching process was carried out using a mixed solution of phosphoric acid (6 wt %) and potassium dichromate (1.8 wt %). The second anodization was conducted under identical conditions as the first but for a longer duration of 8 h. Subsequently, pore-widening was achieved by soaking the sheets in 5 wt % phosphoric acid at 30 °C for 30 min. The sheets were then floated on the surface of a 5 wt % sodium hydroxide solution for 20 min to remove one side of the aluminum oxide layer. Finally, the samples underwent immersion in a copper chloride hydrochloric acid solution to eliminate the aluminum layer. This step resulted in the production of double-opened porous AAO templates without an aluminum layer, featuring an average pore diameter of ∼53 nm and a length of ∼8.15 μm after rinsed with DI water.

### Preparation of the Breath Figure PAzo Films

A 4 wt % PAzo solution in chloroform was prepared initially. Subsequently, the PAzo solution was drop-cast onto glass substrates. The samples were then placed into a sealed container containing boiling water, which was positioned in an oven of 30 °C. After 40 min, the samples were retrieved from the container, resulting in the formation of breath figure PAzo films.

### Preparation of the Hierarchical PAzo Structures via the Light-Induced Nanowetting Method

An AAO template was initially placed on top of a breath figure PAzo film. Subsequently, following exposure to 700 mW of UV light (365–375 nm) for 3 min, the nanopores of the AAO templates were infiltrated by the PAzo. Finally, after the templates were removed using a 5 wt % sodium hydroxide solution and rinsed with DI water several times, the hierarchical PAzo structures were obtained.

### Structure Analysis and Characterization

The chemical structure of the PAzo was determined using a nuclear magnetic resonance (NMR) spectrometer (JEOL 400 MHz). The absorption spectra of the PAzo solution (300–600 nm) were measured using an ultraviolet–visible (UV–vis) spectrometer (Hitachi U-4100). The glass transition temperatures of the PAzo were determined using a differential scanning calorimeter (DSC, TA Q200). DSC measurements were conducted under an N_2_ atmosphere from −50 to 150 °C with heating and cooling rates of 10 °C min^–1^. The morphologies of the samples were characterized using a scanning electron microscope (JEOL JSM-7401F) at an accelerating voltage of 5 kV. Prior to SEM imaging, the samples were coated with 4 nm of platinum using a sputter coater (JEOL JFC-1600) at 20 mA for 50 s to enhance the conductivity. Quantitative analysis of the SEM images was carried out using ImageJ software.

## Supporting Information Available

^1^H NMR spectrum of the PAzo; cross-sectional view SEM images of the breath figure PAzo films under different illumination durations; UV–vis spectrum of the AAO template; SEM images of hierarchical PAzo structures fabricated using AAO templates with different pore sizes (PDF)
