# Supplementary material for: Light-Assisted Fabrication of Hierarchical Azopolymer Structures Using the Breath Figure Method and AAO Templates
Source: Langmuir. 2024 Jul 16;40(30):15941–8. doi: 10.1021/acs.langmuir.4c02410 (PMC11295177; doi:10.1021/acs.langmuir.4c02410)
Supplement: Supplementary file 1 — la4c02410_si_001.pdf [file la4c02410_si_001.pdf]

Supporting Information

Light-Assisted Fabrication of Hierarchical Azopolymer Structures Using the Breath Figure Method and AAO Templates

Ming-Hsuan Chang,[1] Lin-Ruei Lee,[1] Meng-Ru Huang,[1] Tsung-Hung Tsai,[1] Yi-Fan Chen,[1] Yu-Ting Hong,[1] Yu-Chun Liu,[1] and Jiun-Tai Chen[1,2]*

[1]Department of Applied Chemistry, National Yang Ming Chiao Tung University, Hsinchu, Taiwan 300093

[2]Center for Emergent Functional Matter Science, National Yang Ming Chiao Tung University, Hsinchu, Taiwan 300093

*To whom correspondence should be addressed. E-mail: jtchen@mail.nctu.edu.tw. Tel.: +886-3-5731631

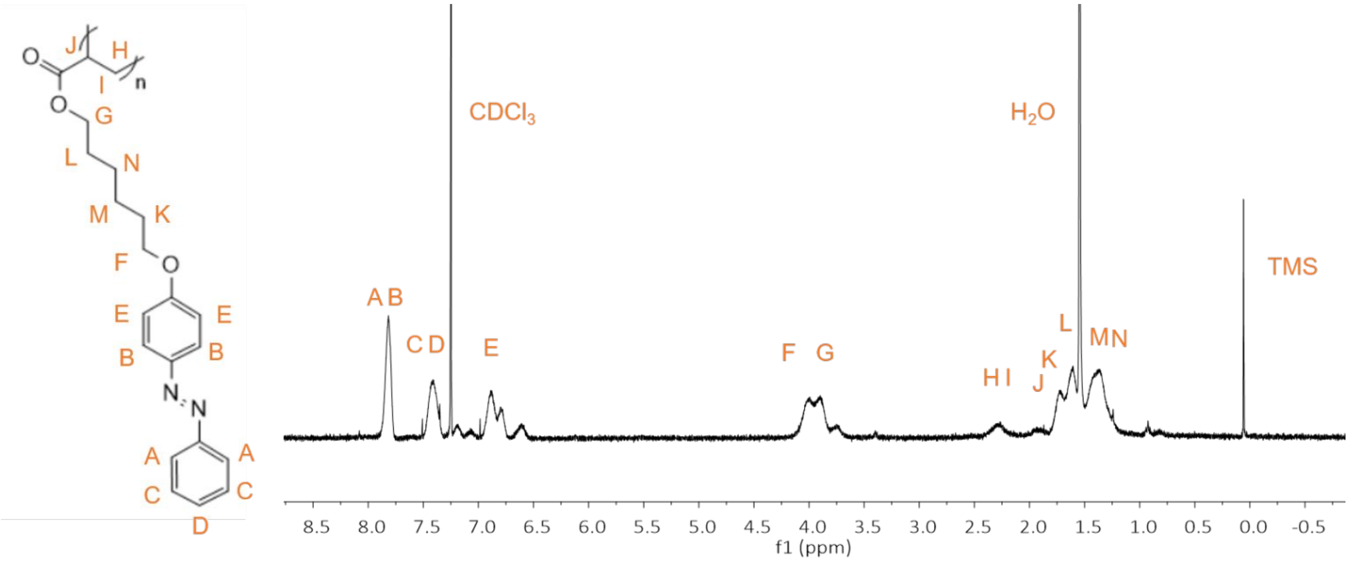

Figure S1. ^{1}H NMR spectra of the PAzo.

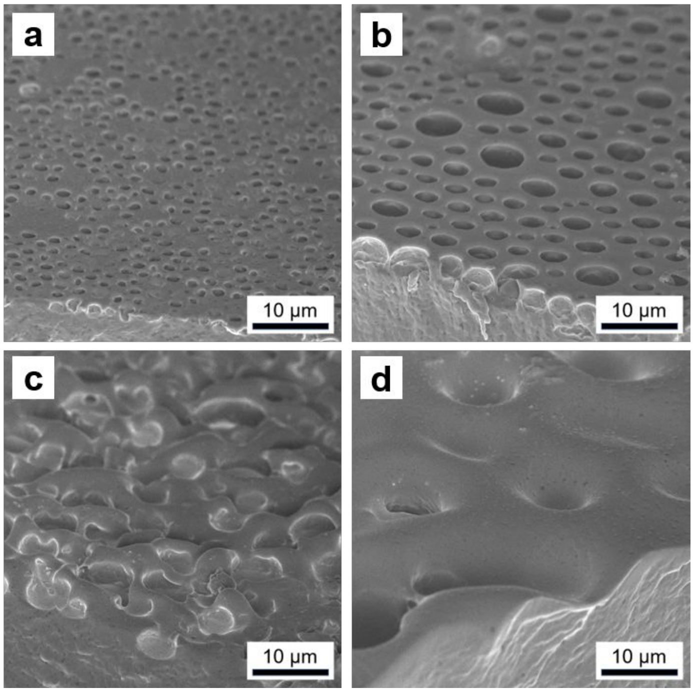

Figure S2. Cross-sectional view SEM images of the breath figure PAzo films under different illumination durations: (a) 0, (b) 10, (c) 20, and (d) 30 min.

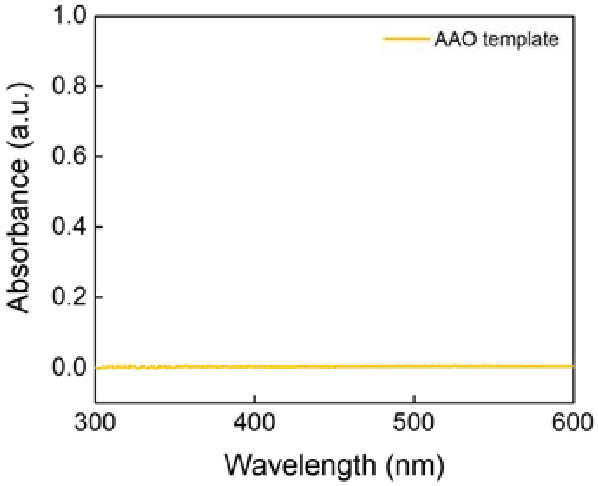

Figure S3. UV-vis spectrum of the AAO template.

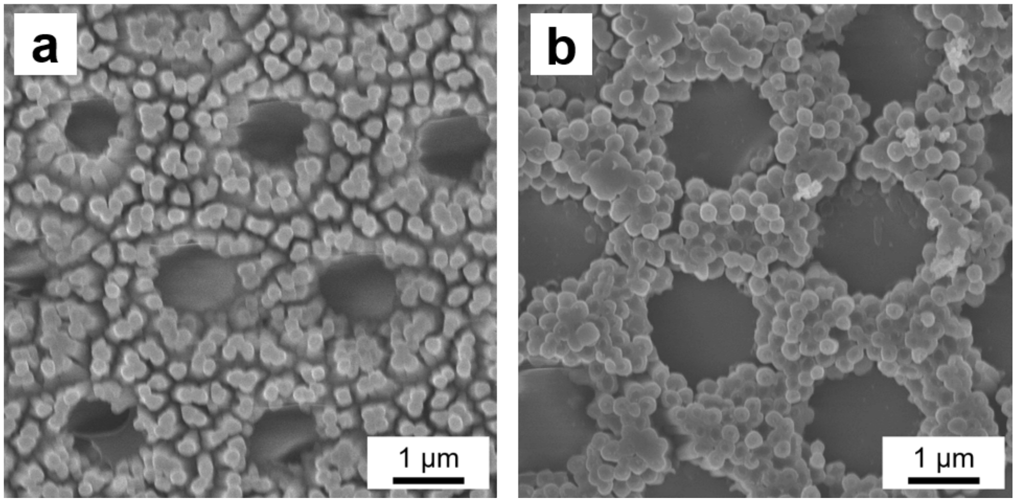

Figure S4. SEM images of hierarchical PAzo structures fabricated using AAO templates with different pore sizes: (a) 100 and (b) 200 nm.
